# Refining Liver Biopsy in Hepatocellular Carcinoma: An In-Depth Exploration of Shifting Diagnostic and Therapeutic Applications

**DOI:** 10.3390/biomedicines11082324

**Published:** 2023-08-21

**Authors:** Zeno Spârchez, Rareș Crăciun, Iuliana Nenu, Lavinia Patricia Mocan, Mihaela Spârchez, Tudor Mocan

**Affiliations:** 1 Department of Gastroenterology, “Prof. Dr. O. Fodor” Regional Institute of Gastroenterology and Hepatology, 400162 Cluj-Napoca, Romania; zsparchez@gmail.com (Z.S.); iuliana.nenu@gmail.com (I.N.); ioanmocan1@ubbcluj.ro (T.M.); 2 Department of Internal Medicine, “Iuliu Hațieganu” University of Medicine and Pharmacy, 400162 Cluj-Napoca, Romania; 3 Department of Physiology, “Iuliu Hațieganu” University of Medicine and Pharmacy, 400006 Cluj-Napoca, Romania; 4 Department of Histology, “Iuliu Hațieganu” University of Medicine and Pharmacy, 400349 Cluj-Napoca, Romania; trica.lavinia@umfcluj.ro; 5 2nd Pediatric Department, “Iuliu Hațieganu” University of Medicine and Pharmacy, 400124 Cluj-Napoca, Romania; mihaelaspirchez@gmail.com; 6 UBBMed Department, Babeș-Bolyai University, 400349 Cluj-Napoca, Romania

**Keywords:** hepatocellular carcinoma, liver biopsy, precision medicine, personalized medicine, therapy, liquid biopsy

## Abstract

The field of hepatocellular carcinoma (HCC) has faced significant change on multiple levels in the past few years. The increasing emphasis on the various HCC phenotypes and the emergence of novel, specific therapies have slowly paved the way for a personalized approach to primary liver cancer. In this light, the role of percutaneous liver biopsy of focal lesions has shifted from a purely confirmatory method to a technique capable of providing an in-depth characterization of any nodule. Cancer subtype, gene expression, the mutational profile, and tissue biomarkers might soon become widely available through biopsy. However, indications, expectations, and techniques might suffer changes as the aim of the biopsy evolves from providing minimal proof of the disease to high-quality specimens for extensive analysis. Consequently, a revamped position of tissue biopsy is expected in HCC, following the reign of non-invasive imaging-only diagnosis. Moreover, given the advances in techniques that have recently reached the spotlight, such as liquid biopsy, concomitant use of all the available methods might gather just enough data to improve therapy selection and, ultimately, outcomes. The current review aims to discuss the changing role of liver biopsy and provide an evidence-based rationale for its use in the era of precision medicine in HCC.

## 1. Introduction and Rationale

The dogma states that histology is the cornerstone of any cancer diagnosis, providing the ultimate argument for malignancy in any clinical scenario. For decades, the pathology report has been the most decisive step in any cancer battle, regardless of perspective: patient, clinician, or researcher. It defined the critical transition from expectative to action, prompting the first step towards a form of resolution. Nevertheless, the modern approach has challenged this dogma as increasing emphasis is placed on a non-invasive strategy for both diagnosis and treatment. For years, hepatocellular carcinoma (HCC) was the epitome of the non-invasive diagnosis success story. Its distinct perfusional pattern permitted a highly accurate imaging-only diagnosis, thus eliminating the need for biopsy in a large proportion of clinical scenarios [1,2,3]. On the flip side, however, the information gathered through imaging is limited, typically responding to a clinical hypothesis in a dichotomous manner, either by confirming or excluding HCC. While this information did suffice in the era of non-targeted therapy, as no particular features guided therapeutic decisions aside from spatial extension [4], it now appears insufficient as HCC has entered the era of precision medicine, primarily due to the emergence of immunotherapy [5].

Consequently, there is a growing need to characterize the tumors better and allow for a perfect tailor-made match between patient, tumor, and therapy, thus revamping the role of biopsy from a confirmatory tool to a more nuanced instrument for in-depth analysis [6,7]. Moreover, the recent advances in liquid biopsy have led to an overall enthusiasm regarding its potential, as its role in diagnosis and therapy is gradually taking shape [8]. In the context of the field’s recently emerged sense of dynamism, this review aims to critically assess the changing role of liver biopsy (LB) in the diagnostic and therapeutic armamentarium of HCC.

## 2. The Changing Role of Liver Biopsy in Hepatocellular Carcinoma

LB remains the de facto gold standard for liver tumor diagnosis. However, advances in imaging, primarily prompted by the widespread implementation of the LiRADS system [1,2], have allowed for precise diagnosis based on sequential imaging methods (computed tomography (CT) and magnetic resonance imaging (MRI)) and therapy commencement without histological confirmation. Consequently, according to the most recent clinical practice guidelines, most liver nodules with particular HCC traits require no histological proof if they occur in a cirrhotic liver. Therefore, all nodules above 20 mm and most ranging between 10 and 19 mm with a highly specific enhancement pattern (LiRADS 5) are eligible for treatment commencement solely on imaging grounds [3,9]. Moreover, the dawn of the imaging-only era in the diagnosis of HCC, which commenced following the first LiRADS report in 2013, overlapped with the latter days of non-targeted therapy in HCC, with treatment being assigned irrespective of specific tumoral biological traits. Given the specificity of diagnostic imaging and the scarcity of options for systemic therapy, with sorafenib as the main staple (a multi-kinase inhibitor not linked to specific gene/receptor expression patterns), the need for biopsy further decreased since it brought no clinically relevant information.

However, oncology has shifted toward a personalized approach, and the era of precision medicine has long been initiated for multiple primary cancers. Breast cancer is probably the most illustrative example, with treatment selection depending on HER2, estrogen, progesterone receptor status, and, more recently, on PD-L1 and *PIK3CA* [10]. Hence, histology is needed for therapy selection irrespective of a positive imaging diagnosis, as a positive “breast cancer” diagnosis bears little consequence for treatment and prognosis without a more in-depth analysis. In this light, HCC was arguably left behind and is only recently entering the realm of personalized therapeutic approaches. At least part of the explanation is the lack of tissue samples in advanced disease to study mutations and tumor biology [11]. The need for tissue sampling to promote research and allow for tailored therapies has been further reinforced by the 2021 American Association for the Study of Liver Diseases (AASLD) consensus on trial design and endpoints in HCC [12], which highlights the potential drawbacks of non-invasive HCC diagnosis. Recent studies on the genomic characterization of HCC have revealed numerous actionable targets for which inhibitors are already available, such as the WNT signaling pathway, immune checkpoint proteins such as PD-1/PD-L1 and CTLA-4, and targets such as VEGFA, MCL-1, IDH-1, TERT, MET, or MDM-4 [13,14]. Acting on the information derived from tumor biology analysis can help better understand treatment response, stratify prognosis, alter therapeutic decisions for trial enrollment, and hopefully improve regimens in the foreseeable future.

Consequently, biopsy in HCC might refine its role from a tool used purely for diagnostic confirmation to a gateway towards precision medicine, expanding the realm of therapeutic possibilities.

## 3. Past and Present: A Biopsy for a Positive Diagnosis in Clinical Practice

An in-depth discussion of the current performance and various LB techniques is beyond the scope of the current review. However, when discussing the changing role of LB in the management of HCC, one must consider the key statistics, figures, and observations from the past decades of clinical use. Unlike most other cancers, pathology in HCC in clinical practice has a sole, straightforward aim—to confirm or reject a positive diagnosis without the burden of additional characterization. Moreover, given that HCC arises almost exclusively in patients with evidence of underlying liver disease (>95%), most often in the setting of a high-risk condition (>87% cirrhosis) [15,16], the pre-test probability of a positive diagnosis is strikingly high. In this setting, in corroboration with previously acquired imaging criteria, the role of biopsy has been restricted to nodules with intermediate risk of HCC and a relatively higher probability of an alternate diagnosis (namely LiRADS-4, LiRADS-M, and LiRADS-NC) [17]. Consequently, LB has a diagnostic yield exceeding 91% [18,19,20], with the accuracy rate of LB increasing with the pre-test imaging probability provided by the LiRADS system [18]. These figures appear not to differ substantially depending on technique (coaxial vs. non-coaxial core biopsy) [21] or timing (first vs. second biopsy after initial non-diagnostic LB) [22]. Not least, even fine-needle aspiration cytology has acceptable accuracy (>80%), with the advantage of using significantly thinner needles [23]. Despite LB’s overall good diagnostic performance, facing the method’s limitations remains clinically consequential. From a diagnostic standpoint, the most relevant is a false-negative result. This instance might occur in up to 30% of the cases and most frequently leads to a second biopsy, thus exposing the patient again to the procedural risks, with a similar diagnostic yield for the second biopsy [22], the most common cause being insufficient tissue sampling (in up to 15% of the cases) [24].

The effectiveness of the imaging-only diagnosis, with a reserve role for LB in scenarios of intermediate HCC risk profile (either by imaging or underlying liver disease), has led to an efficient diagnostic algorithm for HCC, perfectly adapted to the pre-immunotherapy treatment algorithm [4]. Moreover, whenever the non-invasive criteria were insufficient, LB provided a safe alternative with relatively low adverse event rates. The most feared clinically significant complications were bleeding and seeding, accounting for 2–3% of cases, according to various reports [21,25,26,27]. However, bleeding rarely required additional therapeutic measures, seeding appeared not to alter the prognosis [25,26], and the overall procedural-related mortality remained as low as 0.2% [26]. Hence, the guideline recommendation supporting LB whenever needed seems perfectly justified [9].

Another limitation of LB is frequently overlooked when discussing the method’s diagnostic performance: the feasibility of performing the biopsy. Abstaining from LB for various reasons is not typically considered when discussing the diagnostic yield, as most reports only include the cases in which the procedure has been performed. A recently published study by an Italian group that evaluated the feasibility of LB in atypical liver nodules reported a feasibility rate for LB in such nodules below 50%. The factors influencing the feasibility rating were small nodule size (<20 mm), location (segments I and VIII, posterior, centrohepatic, or deep behind a fictitious line of the portal axis), lack of arterial hyperenhancement, and poor inter-observer agreement based on experience [28]. These limitations can be mitigated using real-time contrast-enhancement guidance or sectional imaging fusion techniques [29]. However, these methods are typically used in expert referral centers, and high-quality evidence for broader applicability is currently lacking.

The flipside of the aforementioned diagnostic algorithm is, unfortunately, generated by its most valued attribute: simplicity. Hence, while extremely effective in providing a diagnosis, it lacks the mechanism to provide additional actionable information. A retrospective study performed by an Italian group analyzed the pathological features from the resection specimens of 186 HCC nodules previously classified as LiRADS 3, 4, and 5 [30]. Given that approximately one-third of the nodules were LiRADS 3/4, the study provides valuable information from nodules that are not typically operable. The results demonstrated that LiRADS 5 nodules had a significantly higher rate of microvascular invasion, satellitosis, or capsular invasion. However, tumor grade did not differ significantly, nor did the major outcomes: overall survival, disease-free survival, or cancer-related death. Consequently, encompassing all of the specific enhancement patterns of HCC might not necessarily be associated with a histology subtype or a more advanced tumor grade. Thus, the perspectives for establishing a relationship between previously defined imaging criteria and various HCC phenotypes are less encouraging using conventional imaging methods, leaving the field wide open for other emerging techniques. One such approach might be through radiomics, which could quantify tumor heterogeneity and predict tumor biology, molecular profiles, post-therapy response, and outcomes [31]. A multifaceted diagnostic approach using both imaging and biopsy when required appears sensible, with the ultimate goal of gathering as much consequential information as possible to increase the odds of a favorable outcome.

## 4. Percutaneous Liver Biopsy in Clinical Research: (Re)Defining the Standards

The performance metrics of liver biopsy in the era of precision medicine, specifically in clinical trials, are significantly lower than current clinical use. While diagnostic accuracy of 90% is expected in a clinical setting where a qualitative assessment of malignancy type is sufficient, sample adequacy appears to be astoundingly low for research-specific needs. When sufficient samples were needed for genetic, proteomic, and metabolomic profiling in a clinical trial scenario, sample adequacy dropped to 41.1%, requiring the expansion of the protocol and extending the trial length, even though the protocol used coaxial CB with a median of eight cores per procedure [32]. A potential contributor to these low figures might be the variability of malignant tissue within each core, which was at a median of 40% with an IQR of 10–75%. Furthermore, the study hinted that the HCC subtype might influence biopsy quality, as clear-cell HCC had a 75% tumor load per core vs. a dismal 20% for steatohepatitic HCC. Of note, the differences were not statistically significant due to the relatively small number of cases in each subtype.

Another study, which included both liver and lung biopsies performed in clinical trial settings, reported more favorable figures. Including a total of 76 biopsies assessed, 89.5% were deemed adequate by the investigators, with the most common reason for inadequacy being low RNA yield for sequencing. As a caveat, though, the study did not report an in-depth analysis of the biopsy samples or the specific trial protocol [33].

The role of transitioning liver biopsy from a confirmatory procedure to a tool for personalized medicine might also come with the need for procedure refinement, as better material is required for extensive analysis. There are variations in requirements depending on the research protocol, which are not yet clearly defined. While next-generation sequencing typically requires 10% tumoral tissue, proteomic and metabolomic profiling require in excess of 50% [32]. Consequently, biopsy quality criteria should be conventionally defined to allow for research standardization. The process should ideally start at the bedside with an adequate visual assessment of the cores and be finalized in a feedback loop with the pathology laboratory and trial investigators. Potential variables for calibration might start with defining the optimal number of cores for research purposes, core length, tumor load per core, total cell number, or mass yield [34,35].

## 5. The Role of Histopathology in the Era of Precision Medicine—The Role of Diversity in Hepatocellular Carcinoma

While subtype classification did not bear particular significance in the prior sorafenib-dominated era, when early to intermediate tumors were treated with loco-regional therapies and advanced tumors lacked therapeutic alternatives, the emergence of precision medicine in HCC should increase the emphasis on histological subtypes and more nuanced characterization of each tumor [12].

There is a wide diversity of traits encompassed in the broad term hepatocellular carcinoma, which is far from a homogeneous entity. The only method to adequately distinguish between the subtypes is histopathology. There are three conventional types of cellular organization patterns in HCC: microtrabecular (most frequently encountered), dense or pseudo-glandular (combined in up to one-third of cases), and macrotrabecular (relatively rare) [36]. The latter appears to lead to a recently described clinically significant subtype, macrotrabecular-massive, appearing on surgical specimens as predominantly macrotrabecular (>50% of the tumoral mass), with trabeculae delimited by thick (>6 cells) walls. This phenotype is more often associated with viral hepatitis B and appears to generate a more aggressive disease course, with a higher rate of satellite nodules and vascular invasion [37]. From a genetic standpoint, it is associated with the overexpression of angiopoietin 2 and vascular endothelial growth factor A (VEGF-A), with frequent *TP53* mutations or *FGF19* amplification [38].

The *CTNNB1*-mutated HCC subtype is at the relative opposite end of the severity spectrum. This phenotype is typically well-differentiated, microtrabecular, and lacks peri- and intra-tumoral immune infiltration [38,39]. Another subtype of well-differentiated HCC is steatohepatitic HCC, which is most frequently encountered in cirrhotic and non-cirrhotic non-alcoholic fatty liver disease as well as in alcoholic liver disease. This form is characterized by an exacerbation of the traits of steatohepatitis and implies the presence of Mallory bodies, cellular ballooning, and abundant inflammation in the tumoral microenvironment [40]. Another form associated with a relatively favorable outcome is lymphoepithelioma-like HCC. Despite being poorly differentiated, this subtype has a dense peritumoral lymphocytic infiltrate and a high rate of PD-1/PD-L1 expression, suggesting a potentially effective anti-tumor immune response to PD-1 inhibitors [41].

Moreover, HCC subtypes also harbor cholangiocarcinoma (CCA) traits, namely scirrhous HCC and combined HCC-CCA. Scirrhous HCC is characterized by a thick fibrotic stroma containing islets of malignant cells and has a predominantly progenitor-like expression profile, including CD133, CK7, CK19, and THY1 [42]. The pure HCC-CCA subtype is a particular form displaying full-blown traits of both HCC and CCA, with some arguing that it should instead be classified as a CCA subtype [43].

Furthermore, the spectrum of HCC types is composed of numerous other subtypes, some rare (progenitor HCC), some associated with distinct profiles (fibrolamellar HCC), and some not yet fully coined (sarcomatoid [44], chromophobe with abrupt anaplasia [45]).

To this point, this vast heterogeneity has little to no impact on conventional prognostic stratification or therapy, as the amount of data available for each subtype is scarce and the quality is low. The non-invasive diagnosis era of HCC has generated a unique skew in the available data. On the one hand, there is essential information gathered from resection specimens and explanted livers. These specimens belong to the very early, early, and rarely intermediate stages of HCC, as only these stages are suitable for surgery and seldom require complementary therapy. On the other hand, most of the intermediate (typically treated by interventional therapies) and advanced stages (the main indication for systemic therapy) lack in-depth molecular characterization, leading to a discrepancy in prognostication and treatment response in the stages in which it is needed the most.

Increasing tissue availability by performing liver biopsy, especially in research designs, might provide the resources for these entities’ in-depth genomic, transcriptomic, epigenetic, and proteomic characterization, thus paving the way for personalized medicine in HCC. The jury is still out on whether biopsies can provide good discrimination between the HCC phenotypes. Unfortunately, most of the available data comes from resection specimens or explanted livers. The main obstacle is the relatively high intra-tumoral heterogeneity, as cores from a single tumoral locus may not provide the entire picture. One potential solution might be to use tissue and liquid biopsy hand-in-hand to compensate for the tissue selection bias. We will further expand on the topic in the subsequent paragraphs.

## 6. Biopsy and Precision Medicine in HCC: The Link from Identified Target to Clinical Translation

Most of the tumoral heterogeneity in HCC depends on two main factors: the specific mutational profile and the immune status of the tumor. These are potential therapeutic targets, provide the basis for the current standard of care, and are readily identifiable on pathology specimens. According to the most recent BCLC algorithm [5], all approved first- and second-line therapies target either the checkpoint inhibition pathway, protein kinases, angiogenesis, or cell proliferation pathways. These regimens and their respective target pathways are depicted in Table 1.

However, despite the well-defined pharmacological mechanisms of the available drugs, to this point, there are no clear-cut recommendations for tailored drug selection and pre-therapeutic testing, as in melanoma, colorectal, lung, or breast cancer. In addition, while the most recent updates from the IMbrave 150 trial show substantial increments in overall survival (19.2 vs. 13.4 months) for atezolizumab+bevacizumab compared to the prior standard of care (sorafenib) [46], the figures are still far from the results obtained in other cancers, such as malignant melanoma (exceeding 72 months) [47]. Moreover, the prevalence of some of the targeted mutations is relatively low, which might explain the relatively low survival gains conferred by the currently available drugs. Beyond these already targeted pathways, a wide array of genetic mechanisms are altered in HCC, with at least 30 commonly described mutations [48]. Six genetic alterations are potentially targetable with FDA-approved therapies, while eight are currently in phase I or II testing with variable results. Proof-of-concept studies for some pathways, such as *CTNNB1* using proteolysis-targeted chimeras [49], the IGF pathway [50], TGFβ signaling in NASH-associated HCC [51], or oxidative stress [52], have shown some promise. A list of the most common mutations, their prevalence, targetable status, and our subjective assessment of their translation potential based on prevalence and preliminary testing is provided in Table 2.

Based on the ongoing clinical trials (Table 3), the future of systemic therapy in HCC appears to be composed of multiple-target regimens consisting of either checkpoint inhibitors and targeted therapies or checkpoint inhibitor combinations. In this light, given the multitude of targets and the expansion potential of the therapeutic armamentarium, a precise tumor characterization, similar to the protocols for breast or lung cancer, might pave the way for personalized regimen selection in both first- and second-line scenarios [53].

## 7. Liver Biopsy and the Future of Prognosis and Therapy

Prognostication and treatment selection in the future might go beyond the classifications above or even circumvent them entirely. An artificial intelligence (AI) model based on single-cell spatial analysis and the immune microenvironment identified three reproducible histological subtypes with distinct pathology characteristics, each associated with somatic genomic alterations and specific molecular pathways [54]. Another AI model trained on hematoxylin-eosin stains had a 96% accuracy for benign/malignant classification and 89.6% for tumor differentiation, allegedly similar to a pathologist with five years of experience. The same model effectively predicted four mutations with previously proven prognostic implications, namely *CTNNB1, FMN2*, *TP53*, and *ZFX4*, with AUROCs ranging from 0.71 to 0.89 [55].

Advances in genetic assays have allowed for comprehensive transcriptomic analysis on small-sized samples with degraded RNA, ideal for clinical practice use (i.e., formalin-fixed paraffin-embedded biopsies). One such technique is using the NanoString nCounter to detect gene expression using color-coded probe pairs with very high sensitivity [56]. A French study group tested this method in 2022, and their team identified a genetic signature for one of the critical prognostic indicators in HCC: microvascular invasion. According to their research, not only do biopsy samples correlate well with resection specimens (R = 0.97, thus proving an essential proof of concept), but the 6-gene signature was an above-average predictor for microvascular invasion and was associated with overall survival, with a hazard ratio of 2.29 (95% CI 1.03–5.07; *p* = 0.041) [57].

Furthermore, NanoString-derived genetic data from tissue samples can be combined with deep learning models for visual analysis to construct multifaceted predictive models with enhanced predictive capabilities. Such an approach was proposed by Zeng Q et al. [58] and appeared effective in predicting inflammatory and immune genetic signatures from tissue slides, with some models reaching an AUROC of 0.92. Other AI models based on cancer stem cells have been tested for predicting prognosis and therapy response, providing compelling evidence for a viable approach [59]. Combined with prior evidence hinting that immune-related gene signatures can predict response to immunotherapy [60], such endeavors might pave the way for the future of personalized treatment selection in HCC.

Consequently, the role of pathology in the future of HCC treatment and prognostication may be based both on morphology and functional data, tailor-adjusted in a case-by-case manner by various algorithms. However, the transition to clinically useful tools might face multiple roadblocks generated by the lack of open-source algorithms and large-scale external validation. Moreover, as computational pathology is slowly emerging as a subfield, the technical abilities required for a proper understanding and use of the methods are ever-increasing and expand well beyond the conventional training of practicing clinicians. Hence, overconfidence and an inadequate understanding of the methods might harbor significant ethical and pragmatic risks, transforming such models into veritable double-edged swords [61].

## 8. Liquid Biopsy vs. Tissue Biopsy in Hepatocellular Carcinoma

The era of personalized medicine (especially for advanced HCC) has just begun, and individualized treatment might soon become the new normal. In the transitioning period, tissue and liquid biopsies will have to join forces to provide better care for liver cancer patients. Liquid biopsies (LiqBs) refer to the molecular analysis of tumor components released from a solid tumor into biological fluids such as whole blood, plasma, urine, or bile [62]. In this review, LiqB refers to circulating tumor nucleic acids (DNA and RNA), cells (CTC), and exosomes. We will not summarize all the molecules that have been investigated in HCC so far. This has already been done in several reviews published by our team [62,63,64] and others [8]. Therefore, we will emphasize the need for LiqB not to replace tissue biopsy (TB) but rather to complement it in several clinical scenarios.

### 8.1. Liquid Biopsy in HCC Diagnosis

Unlike any other solid tumor, HCC diagnosis mainly relies on imaging, and tissue samples are rarely available [9,65]. Even though TB might confirm the diagnosis in all cases of HCC, there are situations where a liquid biopsy could provide additional insights. For instance, in patients with small HCCs (between 1 and 2 cm), it is often challenging to target these nodules, and the diagnostic performance is modest in the best-case scenario [66]. In this clinical setting, LiqB could provide a novel solution. One study evaluating several surface markers expressed on extracellular vesicles (EV) showed that AnexinV + EpCAM + tumor-associated microparticles precisely diagnosed tumor nodules between 1 and 2 cm as HCC; the smallest detected nodule was 11 mm in diameter [67]. TB is also challenging in poorly visible or invisible HCC nodules. Contrast-enhanced US, CT, or MRI guidance could be a solution, yet these techniques require special training and are typically accessible only in dedicated tertiary care facilities. One recent study has shown that another combination of EV surface markers (AnnV + CD44v6 + EVs) and AFP could discriminate between HCC and cholangiocarcinoma with 100% sensibility and specificity [68]. Further large-scale confirmation of these results is necessary and could facilitate the transition of LB from bench to bedside. Therefore, LB might take the main stage in patients where TB is challenging or counter-indicated (e.g., small HCC nodules, poorly visible or invisible HCC nodules, patients with coagulation abnormalities, presence of ascites, etc.).

### 8.2. Liquid Biopsy in HCC Prognosis

Personalized medicine in liver cancer patients implies access to genomic data on a patient-by-patient basis, which requires biopsy or surgical specimens for tissue samples of the tumor. TB is an invasive procedure, with bleeding and seeding as significant complications [66]. Furthermore, there are concerns that TB cannot depict the whole spectrum of hepatocarcinogenesis. Both spatial (intra-tumoral) and temporal (changes that occur in the tumor after treatment) heterogeneity have been described in HCC [69]. LiqB rather than TB might capture spatial heterogeneity better [8]. In HCC, a pilot study suggested that detecting mutations in the plasma of HCC patients was feasible and matched the mutations detected in tumor tissue [70]. Several other studies have shown that various circulating DNAs or alterations in circulating DNAs can hint towards early recurrence [71,72,73] or predict overall survival [74,75]. Identifying minimal residual disease is impossible after curative treatment for HCC (especially after tumor ablation). The use of LiqB could overcome this limitation. For instance, circulating DNA (ctDNA) allowed minimal residual disease (MRD) detection in a prospective cohort of 230 patients undergoing surgical resection of stage II colon cancer, findings that might be replicated in HCC. Postoperative detection of ctDNA outperformed prognostic factors such as the TNM stage to predict recurrence-free survival. Furthermore, other molecules, such as circulating free RNA and EVs, were predictive biomarkers in HCC. In-depth analyses of these have been described by others [8,62,63,64]. Temporal heterogeneity is another cancer characteristic that can limit the application of TB. Temporal heterogeneity refers to the molecular modifications in cancer cells after treatment; some cells have acquired the ability to adapt to the new harsh conditions and can emerge in new tumors. Multiple TB in these circumstances is not a solution and should be avoided [25]. LiqB may indeed be an alternative to TB in cases where sequential biopsies are needed. Moreover, LiqB could identify recurrence earlier than imaging, as one study found that mutation detection (in circulating DNA) preceded tumor recurrence as detected by magnetic resonance imaging [73].

### 8.3. Liquid biopsy in HCC Treatment

By far, the systemic treatment of HCC has encountered the most changes in the past few years. Currently, there are multiple options for advanced HCC, and the paradox of choice has rapidly reached the realm of HCC, generating a burden on the oncology community. There is a pressing need for new tools to help stratify patients who will respond to one treatment from those who will not and avoid unnecessary drug reactions. For example, in patients with HCC, von Felden et al. recently demonstrated that mutations of genes from the PI3K/mTOR pathway were predictors of non-response to TKIs, highlighting the potential of LiqB to decide which treatment should be the first-line therapy (i.e., TKIs versus immunotherapy) [76]. Others have shown that phosphorylated ERK (pERK) and pAkt expressions in circulating tumor cells (CTCs) isolated from HCC patients can predict progression-free survival [77]. Once systemic treatment has commenced, it is essential to single out patients with acquired resistance to therapy and switch to alternatives as soon as possible. In a prospective cohort of 42 patients with molecularly defined gastrointestinal cancers and acquired resistance to targeted therapy, direct comparison of post-progression cfDNA versus tumor biopsy revealed that cfDNA more frequently identified clinically relevant resistance alterations and multiple resistance mechanisms, detecting resistance alterations not found in the matched tumor biopsy in 78% of cases [78]. Knowing that circulating tumor cells are more prevalent in intermediate and advanced HCC, we postulate that shortly, CTC isolation from HCC patients and subsequent in vitro testing of these CTCs against various systemic therapies will allow the oncologist to offer the best drug to each patient based on the CTCs’ in vitro resistance profile. In Table 4, we propose some potential indications of LiqB over TB in HCC.

It should be acknowledged, however, that LiqB is still an emergent method with limited high-quality evidence supporting its use in clinical practice. Most of the available data is derived from proof-of-concept studies; the biomarker palette is still deeply heterogeneous, and cut-offs are poorly defined. Moreover, given the lack of standardization and widespread use, LiqB is still relatively expensive from a per-sample standpoint, and cost-effectiveness analyses compared to the current diagnostic gold standard have not yet been performed. Beyond the scope of the current review, an in-depth assessment of the current role of LiqB in HCC diagnosis and prognosis, discussing the promises and caveats of the various techniques, has been elegantly addressed in two recently published articles [8,79].

## 9. So, When Should I Perform a Liver Biopsy? An Evidence-Guided Approach

It is indisputable that LB is again harnessing increasing interest as the era of personalized medicine appears to have finally reached HCC. Consequently, the role of LB has now changed from a confirmatory tool to an instrument of precision. Therefore, expectations have risen, and quality requirements have changed. Based on all the data discussed in the present article, our team has summarized the current perks and pitfalls of LB in Table 5.

On the other hand, the past decade of progress in non-invasive HCC diagnosis must not be ignored, as the LiRADS system provides the ideal framework for an abbreviated diagnosis process and facilitates quicker therapy. Hence, in the setting of a nodule with a definite curative solution and a clear-cut non-invasive diagnosis, increased precision does not translate into improved outcomes, rendering LB useless. One such example might be a BCLC 0/A nodule, LiRADS 5, eligible for surgery.

A more controversial decision might be whether to biopsy lesions, referred to as thermal ablation or trans-arterial chemoembolization (TACE). In the absence of recurrence, especially in the case of thermal ablation, LB might not provide added value. However, in the case of TACE, which has a relatively high post-procedural recurrence rate, LB might provide insights into the prognosis and guide subsequent therapy. This might be particularly important since specific HCC phenotypes have been associated with recurrence after loco-regional therapies [38]. In consequence, a BCLC A nodule treated using loco-regional therapies might not benefit from LB, given its high probability of oncological cure, while a BCLC B nodule might justify a pre-therapeutic biopsy, regardless of the certainty of an imaging-based diagnosis.

Advanced tumors (BCLC C) are the target of systemic therapy and probably represent the clinical scenario in which phenotype, heterogeneity, mutational profile, and gene expression matter the most. While evidence to support this has only emerged in the past few years for HCC, extrapolating data from other cancers predicts an increasing relevance of subtyping and in-depth characterization. Thus, this setting might become the key indication for LB and the place for procedure refinement. Emerging techniques such as LiqB have the ideal scenario for validation and further calibration, as a hand-in-hand journey along with LB might provide sufficient ground to allow for LiqB to leap from bench to bedside. Furthermore, LiqB and LB can perfectly complement each other, with LB offering certainty and validation while LiqB might account for tumor heterogeneity missed by sampling. However, to allow for progress in day-to-day care, consideration should be given to synchronizing research designs to gather sufficiently strong evidence to support progress in clinical practice.

## 10. Conclusions

The current article summarizes the shifting role of LB. Recent evidence has shown that “all liver cancers are alike, but they are alike in a unique way”, paraphrasing Siddhartha Mukherjee’s The Emperor of All Maladies. Consequently, the need to sharpen the available tools to account for the multitude of diseases encompassed by the HCC umbrella is becoming increasingly poignant, as one way or another, HCC appears to be heading back to histology. Therefore, refining LB appears to be the critical gateway to personalized therapy until other promising methods, such as liquid biopsy, fully develop into clinical tools.

## Figures and Tables

**Table 1:** Targeted pathways for the currently approved therapies in hepatocellular carcinoma.

Regimen	Targeted Pathway
First line	
Atezolizumab + Bevacizumab	Anti-PD-L1—immune checkpoint inhibitionVEGF—angiogenesis
Sorafenib	Kinase inhibitors: VEGFR, PDGFR, BRAF
Levatinib	Kinase inhibitors: VEGFR, PDGFR, FGFR, KIT, RET
**Second line**	
Regorafenib	Kinase inhibitors: VEGFR, PDGFR, BRAF
Cabozatinib	Kinase inhibitors: VEGFR, MET, RET
Ramucirumab	VEGFR2
Pembrolizumab	Anti-PD-L1—immune checkpoint inhibition
Nivolumab + Iplimumab	Anti-PD-L1—immune checkpoint inhibitionAnti-CTLA-4—immune checkpoint inhibition

**Table 2:** The clinical translation potential for the most commonly altered molecular pathways in hepatocellular carcinoma (adapted after Llovet J. et al. [48]).

Molecular Pathway	Gene Alteration	Prevalence (%)	Targetable Status	Translation Potential
Telomere maintenance	*TERT*	>50%	In testing	Good
Wnt/β-catenin signaling	*CTNNB1*	10–33%	In testing	Good
VEGF pathway	*VEGFA*	5–10%	Targetable	Good
FGF signaling	*FGF19*	5–10%	Targetable	Promising
Ras/PI3K/mTOR	*PIK3CA*	<5%	Targetable	Promising
	*KRAS*	<5%	Targetable	Promising
	*PDGFRA*	<5%	Targetable	Promising
	*EGFR*	<5%	Targetable	Promising
Cell cycle regulation	*TP53*	10–33%	In testing	Good
	*MYC*	10–33%	No data	No evidence
	*CCND1*	5–10%	In testing	Moderate
TGFβ signaling	*ACVR2A*	<5%	In testing	Moderate
IGF signaling	*IGF2R*	<5%	In testing	Moderate
Oxidative stress	*NFE2L2*	<5%	In testing	Moderate
	*KEAP1*	<5%	In testing	Moderate

**Table 3:** Ongoing and recently concluded phase III clinical trials for systemic therapy in hepatocellular carcinoma.

Trial Identifier (NCT)	Type	Drugs	Target
NCT03298451	Checkpoint inhibitor combination	Durvalumab Tremelimumab	PD-L1 CTLA4
NCT04039607	Checkpoint inhibitor combination	Nivolumab Iplimumab	PD-L1 CTLA4
NCT03755791	Checkpoint inhibitor Targeted therapy	Pembrolizumab Levatinib	PD-L1 VEGFR, PDGFR, FGFR, KIT, RET
NCT03764293	Checkpoint inhibitor Targeted therapy	Camrelizumab Apatinib	PD1 VEGFR2
NCT04344158	Checkpoint inhibitor Targeted therapy	AK105 Apatinib	PD1 FGFR, VEGFR, PDGFR, KIT
NCT03755791	Checkpoint inhibitor Targeted therapy	Atezolizumab Cabozantinib	PD-L1 VEGFR, MET, RET
NCT05904886	Checkpoint inhibitor Checkpoint inhibitor Targeted therapy	Atezolizumab Tiragolumab Bevacizumab	PDL-1 TIGIT VEGF

**Table 4:** Potential application of liquid biopsy and tissue biopsy in hepatocellular carcinoma.

Scenario	Tissue Biopsy	Liquid Biopsy
Diagnosis	In most cases, when clinically necessary	Small HCC (1–2 cm); Poorly visible or invisible HCC nodules; Ascites; Impaired coagulation
Prognosis	At the time of diagnosis	After the initial tissue biopsy—tumoral temporal heterogeneity; Large HCC—tumoral spatial heterogeneity; Detection of minimal residual disease after loco-regional therapies; Detection of recurrence after curative treatment; An alternative to tissue biopsy when multiple or subsequent biopsies are necessary
Treatment	Initial choice of systemic therapy	Initial choice of systemic therapy; During systemic treatment, screen for possible acquired drug resistance; In vitro testing of CTCs for systemic therapies before treatment

HCC—hepatocellular carcinoma, CTC—circulating tumor cells.

**Table 5:** Perks and pitfalls of percutaneous liver biopsy in hepatocellular carcinoma.

Established Advantages	Caveats	Solutions
High diagnostic yield in clinical practice	Disputable diagnostic yield in a research scenario	Increased use of contrast-enhanced guidance; Increased number of cores per biopsy; Improve material selection (needles, core vs. fine needle aspiration); Improve the pathologist–interventional radiologist feedback loop
In-depth characterization of the hepatocellular carcinoma phenotype and molecular profiling facilitate a personalized approach	Might not account for intra-tumoral heterogeneity and post-therapeutic alteration; Lack of extensive evidence that in-depth characterization leads to improved outcomes	Concomitant use of liquid biopsy (as a benchmark); Follow-up using liquid biopsy
Widespread availability	Lack of standardization;	Developing technique-specific protocols and standards of quality;
	Procedural complications (seeding, bleeding)	Method selection; Improved recognition

## Data Availability

No new data were created or analyzed in this study. Data sharing is not applicable to this article.
